# Seismic performance and cost comparison of RC moment resisting and dual frames using UBC 97 and IBC 2021

**DOI:** 10.1038/s41598-024-67373-3

**Published:** 2024-07-16

**Authors:** Sarmad Shakeel, Saadan Hussain Khan, Syed Aayan Saqib, Muhammad Awais Khan, Muhammad Abdul Moiz

**Affiliations:** 1 Department of Civil and Structural Engineering, The University of Sheffield, Sheffield, S1 3JD UK; 2 Institute of Civil Engineering, National University of Sciences and Technology (NUST), H-12, Islamabad, Pakistan

**Keywords:** Building code comparison, UBC-97 vs IBC-21, BCP-21, Pushover analysis, BCP-07 vs BCP-21, Civil engineering, Software

## Abstract

The transition from the Uniform Building Code (UBC-97) to the International Building Code (IBC-21) marked a major shift in the definition of seismic hazard. The term “seismic hazard” in the form of peak ground acceleration (PGA) is replaced by spectral acceleration. This paper investigates the effect of using new seismic hazards on the structural performance of reinforced concrete (RC) buildings. It also looks into the financial impact on the capital costs of new buildings. Useful insights are made to understand the structural performance and financial impact of adopting IBC 21 for structural design in contrast to UBC 97. This study was carried out from the perspective of a developing country, Pakistan. Reinforced concrete moment resisting and dual frames are used as the main structural system of a typical 7-story residential building to investigate the aforementioned effect. The frames are assumed to be located in two locations with high and low seismic hazards. The effect on structural performance is investigated via nonlinear pushover analysis. Financial impact is judged mainly through cost estimation for steel and concrete. A detailed discussion is also presented on the seismic design guidelines in both codes.

## Introduction

In any developing country such as Pakistan, the construction industry plays a massive role in the economy. With time, advancements are being made, and the construction sector must continue to flourish to provide economic and safe solutions for all types of structures. The Pakistan Building Code 2007 (BCP 2007)^1^ was used until the end of 2021 as the main design standard and is now replaced by the Pakistan Building Code 2021 (BCP 2021)^2^. BCP-2007 was originally based on the Uniform Building Code (UBC-97)^3^, whereas BCP-21 is based on the International Building Code (IBC-21)^4^.

There is a significant difference in seismic hazard redefinition between the BCP 2007 and BCP 2021. The former categorized the country into five regions based on peak ground accelerations (PGAs), while the latter introduced short-term and long-term spectral acceleration contour maps, providing more accurate hazard estimates for any site. Moreover, the BCP 2021 introduces seismic design categories, each dictating specific design and building height restrictions. This transition notably impacts the design base shear of buildings, directly influencing their capital cost. The main aim of this paper is to quantify this impact and shed light on how different structural systems adapt to this seismic hazard redefinition.

Several studies have compared structural systems designed under different building codes. Shodolapo et al.^5^ compared building performance under Eurocode 2^6^ and British Standard BS8110^7^, primarily focusing on critical sections such as beams and examining bending moments and shear forces. Rizwan Rashid et al.^8^ analyzed moment-resisting frames (MRFs) and dual systems and concluded that shear walls in structures under 10 stories can render them uneconomical. Although each building has its own specific case, in general, under 10-story buildings perform well even as an MRF structure. The conclusions are derived merely on seismic responses and not through quantity estimation.

Imashi and Massumi^9^ highlighted substantial disparities in shear force determination methods, story drift limitations, and the consideration of vertical force components between Iranian Standard IS 2800-05^10^ and IBC 2003^11^, emphasizing how these differences affect building responses. Nahhas^12^ conducted studies comparing UBC-97 with the Saudi Building Code^13^, showing differences in conservatism and accuracy between these codes. Using a real building case study using the modal response spectral analysis. They concluded that the Saudi Building Code is less conservative than UBC-97 after using base shear values and overturning moments as parameters for comparison. This study is based on using sample buildings from multiple locations in the US. Both the Saudi code and UBC-97 define hazards based on the values of peak ground accelerations. Chebihi and Nasser^14^ compared the Algerian Code^15^, Uniform Building Code 97^3^, and Eurocode 8^16^. The parameters used for comparison are the displacement and base shear values for different soil and strata types. The study shows the difference in displacement and base shear values due to changes in the codes. All three of these codes also define seismic hazards in terms of the PGA. A comparison of IBC 2003^11^ and UBC was also reported by Pong et al.^17^. This is a pivotal study on the subject, with considerable yet inconclusive conclusions. The outcomes for two high-seismic-risk sites in San Francisco and Sacramento are varied. This study revealed that UBC is more conservative in some areas, although the results are not conclusive.

Though most of the studies explained earlier used traditional code based lateral static analysis approaches for seismic performance assessment, there have also been numerous other seismic design and assessment approaches developed in the literature. Stochastic capacity spectrum method (CSM)-based displacement-oriented design strategy^18^ has been applied on precast steel-reinforced-concrete and ultra-high-performance-concrete composite braced-frame, which resulted in much superior performance under different demand levels. Another innovative approach is probabilistic seismic capacity assessment^19^ which has demonstrated effectiveness for bolt-connected steel-plate reinforced concrete buckling-restrained-brace-frame. This method provides comprehensive damage thresholds and integrates fragility frameworks with seismic analyses^20^. Advanced probabilistic seismic fragility analysis approaches, such as least squares regression, maximum likelihood estimation, kernel density estimation, and Monte Carlo simulation, have also been compared in past^20^ to evaluate their effectiveness under non-stationary ground motions. These methods provide insights into the demand distributions and fragility curves, seeking the accurate assessment of seismic vulnerability and performance. Additionally, a consistent seismic hazard and fragility framework, leveraging the probability density evolution method, has been proposed to account for combined capacity-demand uncertainties. This framework integrates within the performance-based approach, offering a robust non-parametric hazard and fragility assessment scheme that enhances accuracy and efficiency without the need for pre-defined fragility shapes^21^.

In a nutshell, non-linear methods like pushover static analysis, emerge as better tools for understanding building behavior compared to linear analysis^22^ for practical purposes. Most studies use responses like bending moments, shear forces, story drifts, response spectrum, and displacements as measures for comparison. Although numerous comparisons between different codes have been done in past, research specifically contrasting UBC 97 and IBC 21 is scarce, despite their significant divergence in seismic design guidelines. This paper aims to bridge that gap, offering insights into the differences between the two codes. Using nonlinear analysis, it predicts building model responses under seismic design scenarios and compares the costs associated with adopting IBC 21 versus UBC 97. This information could be crucial for decision-makers in countries like Pakistan transitioning from UBC 97 to IBC 21. However, this study’s scope is limited to two sites with different seismic hazard levels and the two most used structural systems, MRF and Dual systems.

This paper is organized to give information on structural design of case study buildings in section “Comparison of design capacities” and the comparison of structural design using both codes in section “Nonlinear analysis”. Moving forward, section “Quantity estimation” presents the outcomes derived from the structural analysis conducted, while section “Conclusions” provides a detailed breakdown of the cost estimation results associated with the design. Finally, the paper concludes by summarizing key insights and implications in the last section.

## Structural design of the case study buildings

An existing 7-story apartment building in Islamabad, Pakistan, is selected as a case study building. The architectural plans of the building are used to design a structural system. The building is designed using two different structural systems: the MRF system and the Dual system (MRF + shear walls). The two structures are then assigned seismic load demands according to both building codes, UBC 97^3^ and IBC 2021^4^, to evaluate the effect of the change in the code on the structure’s seismic design. Additionally, both structures are assumed to be located at medium or high seismic hazard locations. The different choices of building locations will help in understanding the effect of the two codes guidelines on the design of seismic hazard levels.

### Building geometry

The building is 150′ × 90′ in plan and has a total height of 71.5′. The 2 structural systems are modeled and designed for 2 different locations with different degrees of seismicity. According to UBC 97 and BCP 2007^1^, seismic hazards are defined in terms of peak ground accelerations (PGAs). Pakistan was divided into different zones based on the value of PGAs. Zone 2B and Zone 3 correspond to the selected high- and medium-risk seismic locations, respectively. These two locations also show apparent changes in their seismic demand as per the new codes BCP 2021^2^ and IBC 2021^4^.

**Figure 1:**
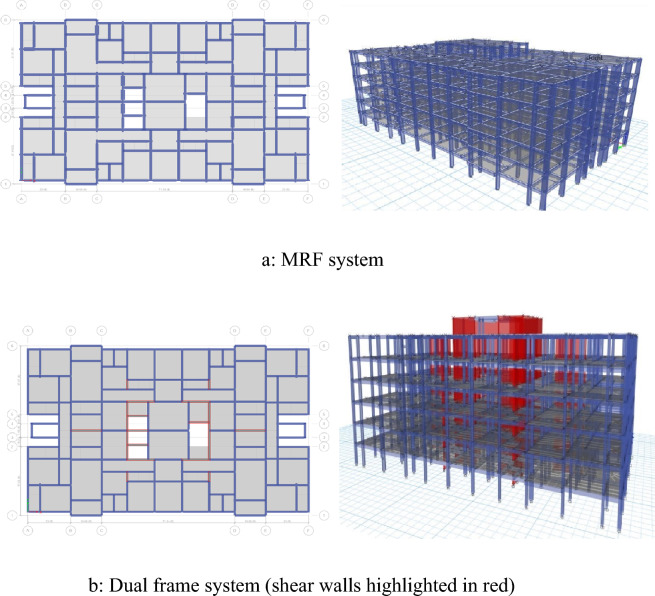
Representations of building models.

ETABs^23^ structural analysis software has been used for modeling purposes. The floor plans of the buildings with both structural systems are shown in Fig. 1. The black dots show the locations of the columns, the blue lines are the beams, and the gray lines show the floor slabs. The white spaces in gray indicate openings in the slab for access to the other floors. The MRF is considered symmetric both in its geometry and stiffness of the columns. This approach avoids the consideration of orthogonal effects and significant torsion in analysis and design. The columns are placed and oriented accordingly to achieve symmetry in the stiffness of the structure. To ensure that the system qualifies as a Moment Resisting Frame (MRF), an initial Equivalent Lateral Force Analysis was conducted. This analysis confirmed that more than 75% of the lateral forces were resisted by frame elements, in accordance with ASCE 7 recommendations. In the Dual frame, similar geometric symmetry is used while there is a combination of both core and planar shear walls in addition to columns. The columns and shear walls stiffness are balanced in a way to achieve equal stiffness in both global directions. To confirm that the system is a Dual System, an initial Equivalent Lateral Force Analysis was performed. This analysis verified that at least 25% of the lateral forces are resisted by frame elements, as recommended by ASCE 7. The slab is 5in deep while the shear walls are 12in thick. Concrete strength of 3000 psi is used while 60 ksi and 40 ksi steel are used for longitudinal and shear reinforcement respectively.

Table 1 shows the different cases of building models developed based on the 2 different structural systems under the two codes UBC 97 and IBC 2021 for two locations, resulting in a total of 8 different models.

**Table 1:** Cases of building models.

Case	Reference design code	Seismic hazard	Structural system
1	UBC 97 (BCP 2007)	Medium: Zone 2B (PGA = 0.16–0.24 g)	MRF system
2			Dual system
3		High: Zone 3 (PGA = 0.24–0.32 g)	MRF system
4			Dual system
5	IBC 2021 (BCP 2021)	Medium: S_s_ = 1.30 g, S_1_ = 0.38 g	MRF system
6			Dual system
7		High: S_s_ = 1.56 g, S_1_ = 0.49 g	MRF system
8			Dual system

### Loading

Both UBC 97 and IBC 2021 use the same gravity load combinations, as listed in expressions (1) and (2). However, combinations involving seismic loads are different for IBC 2021 than for UBC 97. In the lateral (seismic) load combinations, the whole combination is multiplied by 1.1 as per UBC 97 but not as per IBC 2021. Expressions (3) and (4) show the UBC 97 load combinations involving seismic loads for high-seismic-risk sites, while expressions (5) and (6) are the same for IBC 2021.

1 $$1.2{\text{D }} + \, 1.6{\text{L}}$$

2 $$1.4{\text{D}}$$

3 $$1.474{\text{D }} + \, 0.55{\text{ L }} + 1.1{\text{E}}$$

4 $$1.144{\text{ D }} + \, 1.1{\text{E}}$$

5 $$1.3736{\text{D }} + \, 0.5{\text{ L}} + 1{\text{E}}$$

6 $$1.0736{\text{D }} + \, 1{\text{E}}$$

In the above load combinations, D represents the dead load, L represents the live load due to building occupancy, and E is the earthquake load (design base shear). The values for dead and live loads are taken from Pakistan building codes and have remained unchanged between the 2007 and 2021 editions. An equivalent later load analysis procedure is used to define the earthquake load. For the low-seismic-hazard site, the hazard value corresponds to Zone 2B (PGA = 0.16–0.24 g) according to BCP 2007, and the spectral accelerations Ss and S1 are 1.302 g and 0.381 g, respectively, according to BCP 2021. The high-seismic-hazard site can be given as Zone 3 (PGA = 0.24–0.32 g) according to BCP 2007, and the spectral accelerations Ss and S1 are 1.5596 g and 0.4996 g, respectively, according to BCP 2021.

The UBC 97 and IBC 2021 also employ different approaches for the conversion of the vertical component of the earthquake load to equivalent dead loads (Ev). The following equation shows the approach adopted by both codes for computing Ev, where Ca and SDS are seismic design parameters, and I is the importance factor:

7 $${\text{UBC }}97:{\text{ Ev }} = \, 0.5 \, \times {\text{ Ca }} \times {\text{ I }} \times {\text{ D}}$$

8 $${\text{IBC }}2021:{\text{ Ev }} = \, 0.2 \, \times {\text{ SDS }} \times {\text{ D}}$$

This results in different factors of the vertical load in the seismic load combinations for different locations.

In equivalent lateral force analysis, the design base shear is computed by reducing the peak base shear by a factor R, known as the response modification factor. The R factor represents the system inherent ductility and design over strength. In UBC 97, the intermediate moment resisting frame (MRF) system has an R value of 5.5, and the special moment resisting frame (SMRF) system has an R value of 8.5. Their dual systems have R values of 6.5 and 8.5, respectively. In IBC 2021, the IMRF system has an R value of approximately 5, and the SMRF system has an R value of 8. Their dual systems have R values of 6.5 and 7, respectively. The frames are designed as IMRFs or their dual frames in this study only.

### Structural design

All the building case studies are analyzed and designed to obtain the relevant cross-sectional details and steel requirements. The design was carried out with the aid of ETABs software^23^ using both codes. Both UBC 97 and IBC 2021 have seismic detailing requirements for structural members. The gravity design guidelines are based on ACI 318^24^. Suitable cross sections with proper reinforcements are selected for the beams, columns, and shear walls. These cross-sectional sizes are finalized by the hit and trial method, keeping in view the demand on the respective members in different locations of the structure. The sections are chosen to maintain the maximum structural efficiency.

## Comparison of design capacities

This section summarizes the key responses obtained from structural analysis. In particular, the design base shear and story drifts are compared.

**Figure 2:**
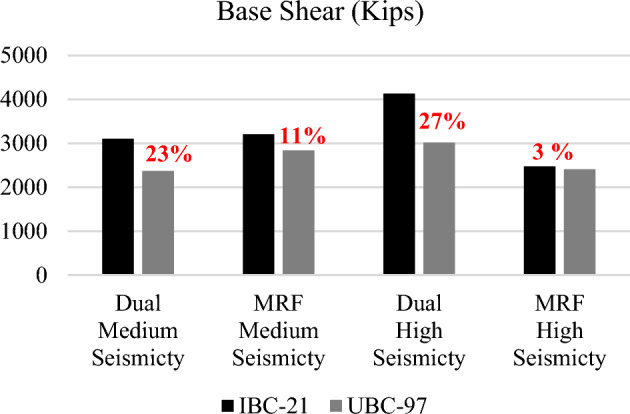
Comparison of design base shears.

Both systems under IBC-21 exhibited greater base shear than those under UBC-97 for both locations/zones. This shows that there is an increased seismic demand for both sites according to the IBC-21 standard. This could be attributed to more detailed hazard estimates for the selected sites available in the new Pakistan Building Code^2^. The Base Shear results are displayed in the form of bar graphs for both codes and locations in Fig. 2. The design base shear for dual systems designed according to IBC-2021 is 23% and 27% greater for medium and high seismic hazard sites, respectively, than for UBC-97. However, the design base shear for MRF systems designed according to IBC-2021 is 11% and 3% greater for medium and high seismic hazard sites, respectively, than for UBC-97.

**Figure 3:**
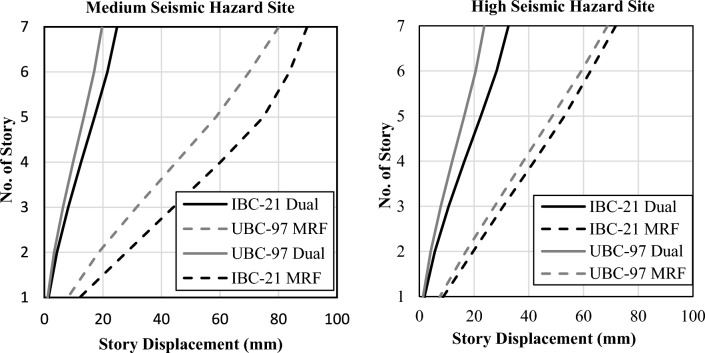
Maximum story displacements.

The maximum story displacements under the seismic design situation are obtained from structural analysis software and are shown in Fig. 3.

**Figure 4:**
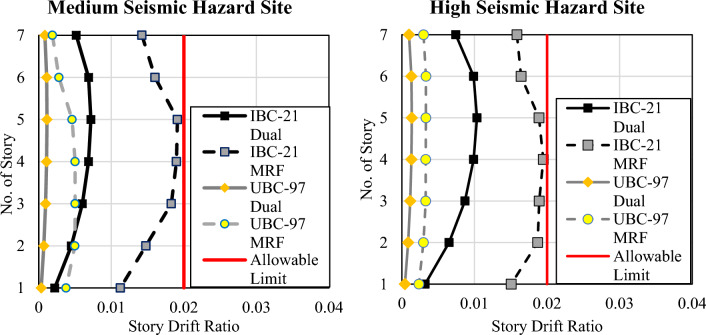
Comparison of interstory drift against code drift limitations.

Both systems designed as per the UBC 97 guidelines showed less displacement than those under the IBC 2021 guidelines for both locations. This can be attributed to the increase in seismic hazard per BCP 2021 or IBC 2021. Compared with MRF systems, dual systems exhibit far less displacement because they offer greater stiffness. To further investigate the results, interstory drifts are also computed and compared with the code requirement for the drift limits, as shown in Fig. 4. A 2% interstory drift limit is proposed for UBC 97 for long-period structures^25^. A similar limit is also identified in ASCE 7-16 Table 12.12-1^26^.

A similar trend is observed for story drifts as that for story displacements. All the story drifts are within the allowable limits of 2% according to both codes.

## Nonlinear analysis

### Nonlinear modeling

Nonlinear analysis of the buildings was primarily performed to investigate the response to different systems designed according to both codes. To simplify the analysis process, only buildings located at high seismic risk are investigated. Hence, a total of 4 models are prepared and analyzed using the two structural systems under both codes.

Nonlinearity is assigned at the member level by defining the stress‒strain curves of the steel and concrete materials used for the columns, beams and shear walls. A rigid diaphragm is assigned to all the slabs. There are two approaches that are used to assign nonlinearity to members, namely, the fiber modeling approach and the plastic hinge modeling approach. The cross-sectional size and the number and layout of the steel bars used are needed as inputs to assign the fibers to the columns and shear walls and plastic hinges to the beams.

The fiber modeling approach is used for assigning nonlinearity to columns and shear walls, whereas plastic hinges are assigned to the beams. A similar approach has been reported in the literature for modeling concrete frame elements^27^. Several detailed models^28^ exist in the literature for the selection of plastic hinge length. However, in this study, hinges are assigned up to 10% of the member length on both sides for the sake of simplification. The fiber hinges used were P-M2-M3 default hinges, and for the beams, M3 default hinges were used as per the instructions in ASCE 41-17^29^. The plastic hinges are assigned nonlinear curves, and the intermediate occupancy (IO), life safety (LS), and collapse prevention (CP) performance levels are assigned to the curves based on ASCE 41-17. Tables 2 and 3 describe the performance limits adopted for concrete (3000 psi) and steel (60,000 psi) in hinges, respectively.

**Table 2:** IO, LS, and CP values for concrete.

Acceptance criteria	Description	Strain (in/in)
Compression		
IO	Onset of compression yielding	0.01
LS	2 times of compression yielding	0.02
CP	5 times of compression yielding	0.05
Tension		
IO	Onset of tensile yielding	0.00088
LS	3 times of tensile yielding	0.0022
CP	5 times of tensile yielding	0.0036

**Table 3:** IO, LS, and CP values for steel.

Acceptance criteria	Description	Strain (in/in)
Compression		
IO	Onset of compression cracking	0.002276
LS	Onset of peak stress	0.004552
CP	Onset of significant strength degradation	0.006828
Tension		
IO	Onset of tensile cracking	0.002276

### Nonlinear performance evaluation

The performance point and the target displacement are calculated by converting the capacity and demand curves under similar variables, and the overlapping point of these two curves is generally considered to be the expected displacement that would be experienced by the structure during a real earthquake, as per the guideline procedures of the Capacity Spectrum Method, specified in ATC 40^30^.

**Figure 5:**
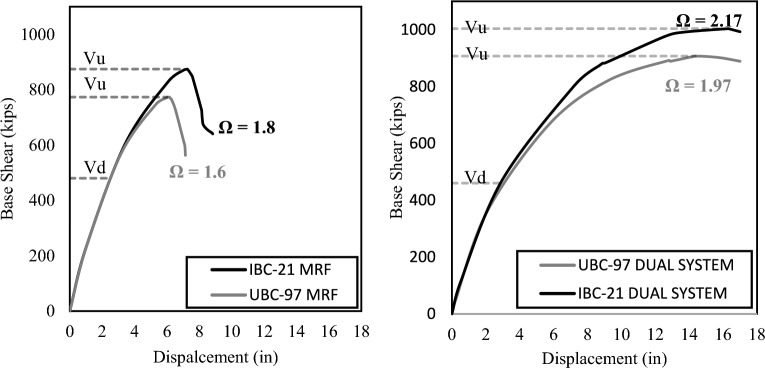
Pushover curves comparing over strength.

Pushover analysis is used to evaluate the structural performance of building case studies beyond the linear range to determine the strength and ductility. The performance of buildings designed as per IBC 2021 is better than that of buildings designed as per UBC 97. The overstrength ratio is the ratio of the maximum base shear of a building to the design base shear. It is a measure of inherent overstrength in a building due to its design. Table 4 and Fig. 5 show the levels of overstrength achieved by the different models. Overall, the buildings designed as per IBC 2021 exhibited higher overall strength than those designed as per UBC 97.

**Table 4:** Overstrength ratio and ductility comparison for buildings at high seismic hazard sites.

	MRF system	Dual system
Overstrength ratio (Ω)		
UBC-97	1.6	1.97
IBC-21	1.8	2.17
Difference	12.5%	10%
Ductility		
UBC-97	2.47	4.95
IBC-21	2.89	5.62
Difference	17%	13.5%

**Figure 6:**
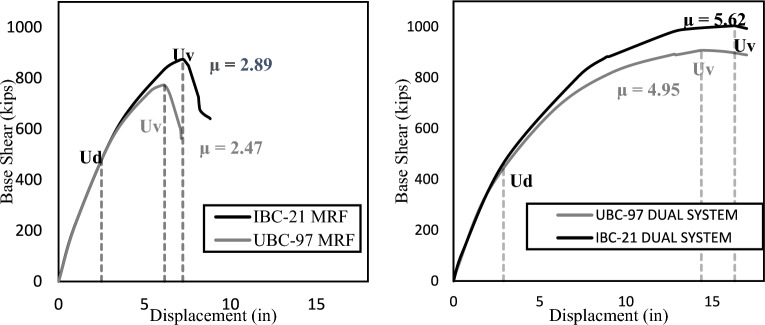
Pushover curves comparing ductility.

The ductility is a measure of the ability of a building to retain permanent deformations before collapsing. Compared with brittle failure, ductile failure is preferred because it allows a reasonable warning time before failure. The ductility is calculated as the ratio of the drift at the maximum base shear (U) to the drift at the design base shear (Ud). The ductility of the structures is also compared, as illustrated in Table 4 and Fig. 6.

As illustrated above in Table 4, there is an increase in the overstrength ratio and ductility in the IBC 2021 models compared to those in the UBC-97 models. The primary reason is the increase in steel requirements. As discussed in the subsequent section, an increase of 15–30% is observed in steel quantities, leading to a more ductile and stronger structure. These results imply that the structures designed according to IBC-2021 are safer than those designed according to IBC-97. This possibly hints at the structural reassessment of existing buildings designed using UBC 97. An increase in the overstrength and ductility is observed more in the MRF system than in the dual system, which implies that the MRF structures are made even stronger as per IBC 2021. This finding also implies that the MRF structures built upon UBC-97 can be more vulnerable to new seismic demands than dual-system structures.

The Pushover Analysis shows that the buildings designed as per IBC 2021 have a greater overstrength ratio and are more ductile in comparison to UBC 97. Pushover analysis also revealed greater differences in the Overstrength and Ductility of the MRF structural system than in the Dual structural system due to changes in the code. The overstrength ratio increased by 12.5% in the MRF system and 10% in the Dual system when UBC 97 was shifted to IBC 2021. However, the ductility increased by 17% in the MRF system and 13.5% in the dual system when UBC 97 was shifted to IBC 2021.

The plastic hinges that developed at the target displacement for each model are illustrated in Fig. 8. The target displacements are 7.2 inches for the UBC-97 MRF structure, 7.54 inches for the IBC-21 MRF structure, 8.35 inches for the UBC-97 Dual Structure, and 8.48 inches for the IBC-21 Dual Structure.

**Figure 7:**
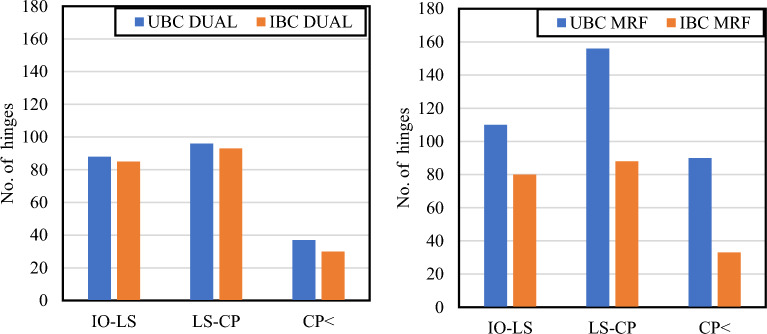
No. of hinges at target displacement.

The number of hinges (Fig. 7) in different performance levels at the target level shows that the UBC 97 MRF model is the most damaged at the performance point. It can also be deduced that compared with the dual system, the MRF has more differences in damage because of changes in the code.

## Quantity estimation

The quantity estimation of all eight models is carried out both manually and with CSI Detail software^31^. Since the comparison is focused on structural systems, only the structural members were considered for quantity estimation.

**Figure 8:**
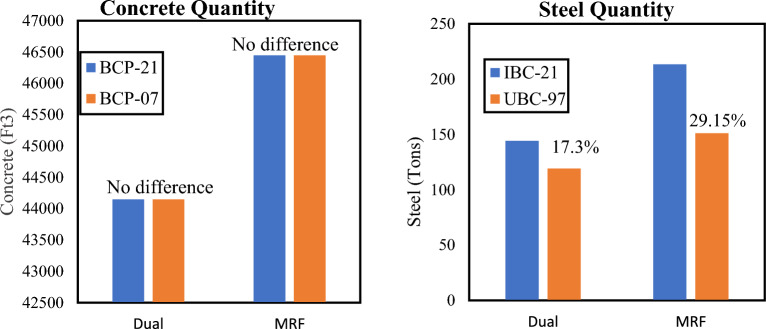
Material quantities for Medium Seismic Hazard Site.

**Figure 9:**
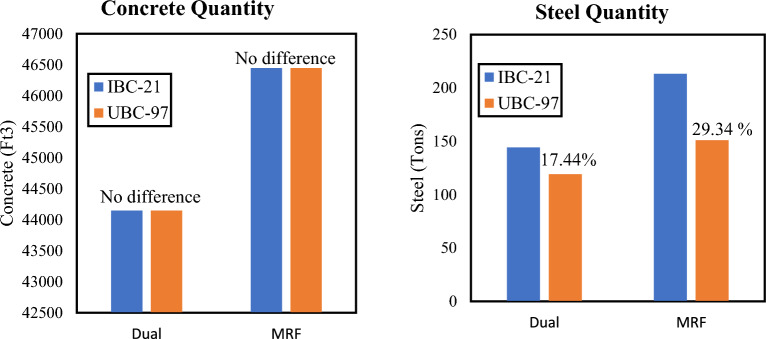
Material quantities for High Seismic Hazard Site.

The quantities for concrete and steel are calculated for each respective model, in cubic feet and tons, respectively. These quantities are used to visualize the difference in the ultimate cost of the structure. These quantities were extracted from beams, columns, shear walls and slabs. The results are plotted in the form of bar graphs in Figs. 8 and 9. Buildings designed as per IBC 2021 always showed higher steel quantities, which is in turn agreement with the stringent code requirements of IBC 2021. The quantities of concrete remained unchanged as the cross-sectional dimensions were kept constant while switching between codes. If comparisons are drawn in terms of structural systems, dual systems are more economical than MRF systems. Under any code, the MRF system has more concrete than does the Dual system. This is because shear walls cause both the columns and beams to experience less stress by attracting seismic forces to themselves due to greater stiffness.

## Conclusions

This paper investigates the effect of using new seismic hazards in Pakistan Building Codes on the structural performance of reinforced concrete (RC) buildings. Reinforced concrete moment resistance and dual frames are used as the main structural system of a typical 7-story residential building to investigate the effect code update (BCP-21 from BCP-07) in Pakistan. The frames are assumed to be located in two locations with high and low seismic hazards. The effect on structural performance is investigated via nonlinear pushover analysis in ETABS software. The financial impact is judged mainly through cost estimation for steel and concrete.

Both systems under BCP-21 show greater base shear and displacements than those under BCP-07 for both locations/zones. This shows that there is increased seismic demand for both medium- and high-seismic-hazard sites, as per the new code of 2021. The difference in base shear is more apparent in Dual systems than in MRF systems. The dual systems showed far less displacement than did the MRF systems. This is because dual systems have greater stiffness than MRF systems, so greater forces are required to displace them.The pushover analysis shows that the buildings designed according to BCP-21 have a greater overstrength ratio, are more ductile and suffer less damage than those designed according to BCP-07. The results also showed that the MRF structural system exhibited greater differences in terms of its overstrength and ductility than did the dual structural system due to changes in the code.

Both systems under BCP-21 have greater quantities of steel (and hence cost) than those under BCP-07. This again verifies that the seismic demand is greater in the new code than in the previous code. In terms of structural systems, dual systems are more economical than MRF systems. Under any code, the MRF system has more concrete than does the Dual system. This is because shear walls cause both the columns and beams to experience less stress by attracting seismic forces to themselves due to greater stiffness.

Nonlinear static analysis and cost comparison done in this study can be significant for the engineering community in countries like Pakistan. They offer better understanding of the differences between both codes. Dual and moment-resisting frame (MRF) systems are among the most widely used structural typologies. The findings presented here provide essential guidance for structural engineers during the design phase, helping them select the appropriate building system. Additionally, the study offers advice on potential rehabilitation interventions for buildings designed using outdated codes. To further strengthen these conclusions, nonlinear dynamic analysis techniques could be employed. It’s important to note that this study is limited to two sites with significant seismic risk. Future work could extend the scope to include more sites with increased seismic risk.

## Data Availability

All data generated or analyzed during this study are included in this published article. Further information on the data can be made available from the corresponding author on reasonable request.
